# Supplementary material for: Combination Ad26.RSV.preF/preF protein vaccine induces superior protective immunity compared with individual vaccine components in preclinical models
Source: NPJ Vaccines. 2023 Mar 23;8:45. doi: 10.1038/s41541-023-00637-7 (PMC10033289; doi:10.1038/s41541-023-00637-7)
Supplement: Supplementary file 1 — Supplementary Information [file 41541_2023_637_MOESM1_ESM.docx]

**
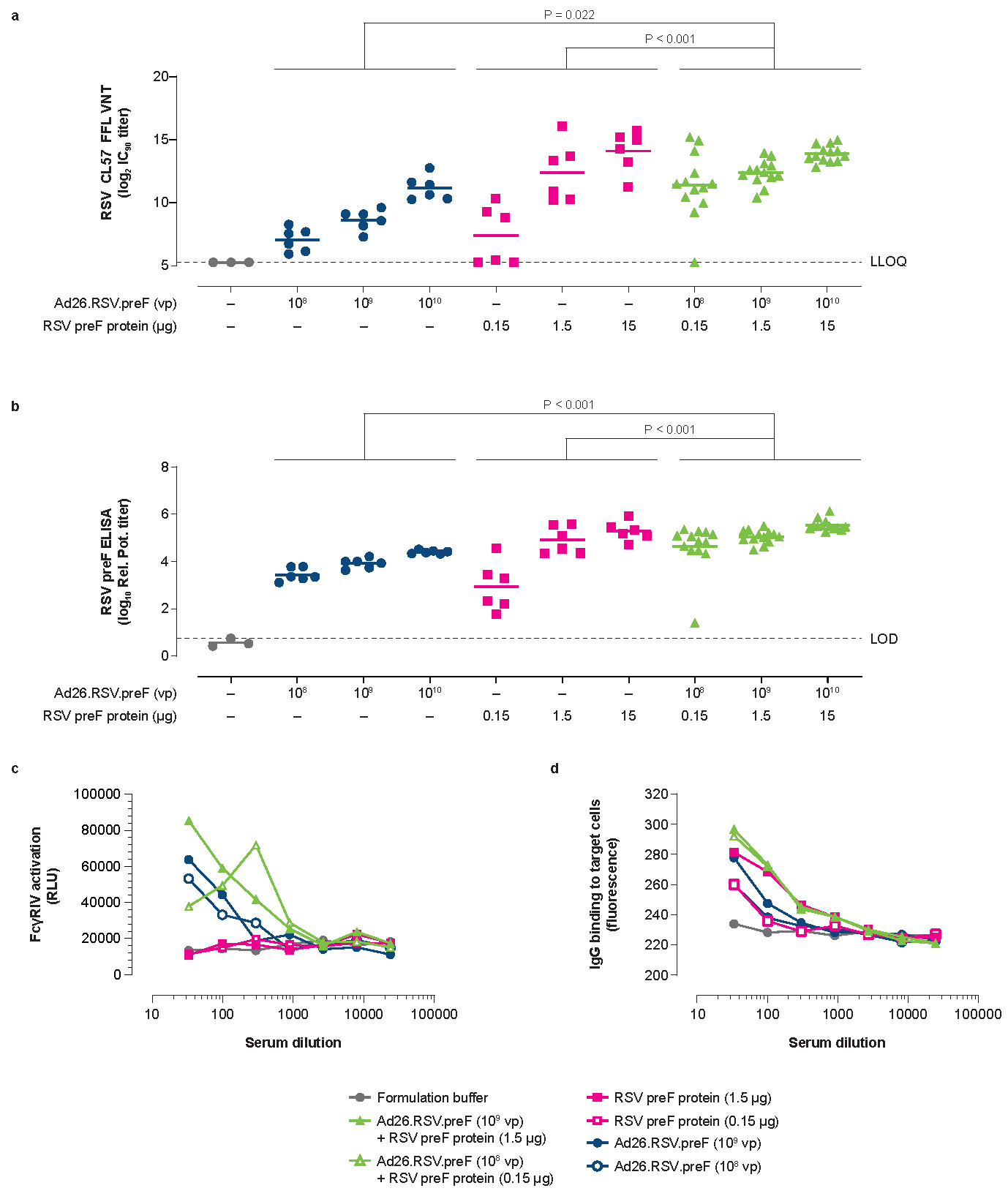
**

**Supplementary Figure 1. VNT and RSV preF-specific IgG responses and FcγR engagement of antibodies induced by Ad26.RSV.preF/RSV preF protein combinations in naïve mice.** Naïve female BALB/c mice were immunized at Week 0 and Week 6 with Ad26.RSV.preF (1×10^8^, 1×10^9^, or 1×10^10^ vp; n = 6 in each group), RSV preF protein (0.15, 1.5, or 15 µg; n = 6 each), or Ad26.RSV.preF combined with RSV preF protein at varying dose levels (n = 13 each). Week 8 serum RSV CL57 VNT (log_2_ IC_90_ titers), measured using FFL virus neutralization assay **(a)** and RSV preF–binding IgG antibodies (log_10_ Rel. Pot. Titers), measured by ELISA **(b)**. Horizontal bars indicate mean group titers; dotted lines indicate the LLOQ (**a**) and LOD (**b**), respectively. LOD was specified as highest titer measured in the formulation buffer control group. Serum obtained at Week 8 from mice immunized with the middle (Ad26.RSV.preF [1×10^9^ vp] and RSV preF protein [1.5 ug], alone or in combination) and lowest (Ad26.RSV.preF [1×10^8^ vp] and RSV preF protein [0.15 ug], alone or in combination) vaccine concentration was pooled and used to measure FcγRIV activation in an ADCC reporter assay (**c**), expressed as RLU. Total IgG binding to RSV F–expressing target cells was measured by fluorescence using AF488-conjugated goat anti-mouse IgG (**d**). Average of duplicate measurements is shown. Ad26, adenovirus type 26; ADCC, antibody-dependent cellular cytotoxicity; AF488, AlexaFluor^®^ 488; ELISA, enzyme-linked immunosorbent assay; FcγR, Fcγ receptor; IC_90_, 90% inhibitory concentration; IgG, immunoglobulin G; LLOQ, lower limit of quantification; LOD, limit of detection; preF, prefusion conformation–stabilized RSV F protein; Rel. Pot., relative potency; RLU, relative light units; RSV, respiratory syncytial virus; vp, viral particles.


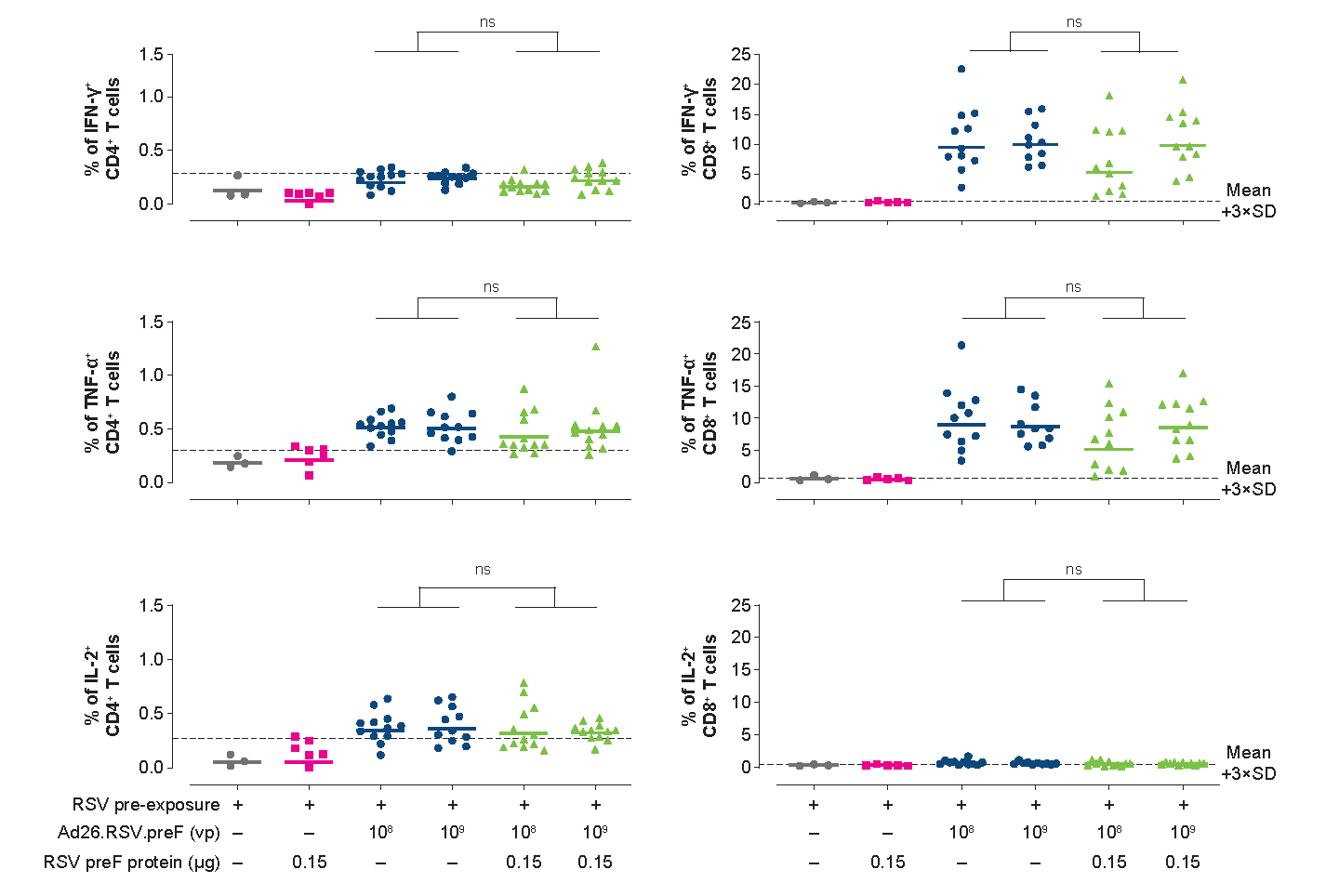


**Supplementary Figure 2. ICS measured at Week 5 in splenocytes isolated from RSV pre-exposed mice.** Female BALB/c mice were pre-exposed i.n. with RSV A2 (5×10^5^ pfu) at Week –79 and immunized intramuscularly at Week 0 with Ad26.RSV.preF (1×10^8^ or 1×10^9^ vp; n = 12 in each group), RSV preF protein (0.15 µg; n = 6), Ad26.RSV.preF (1×10^8^ or 1×10^9^ vp) combined with RSV preF protein (0.15 µg; n = 12 each group). Negative control animals received formulation buffer (n = 6). Cellular responses were measured by ICS in splenocytes at Week 5, and the percentage of CD4+ (left panels) and CD8+ (right panels) T cells expressing IFN-γ (top panels), TNF-α (middle panels), and IL-2 (bottom panels) is shown. The horizontal lines indicate the geometric mean titer per group, and the dotted line indicates 3×SD above the mean response of unstimulated cells. Ad26, adenovirus type 26; ICS, intracellular cytokine staining; IFN-γ, interferon-γ; IL-2, interleukin-2; i.n., intranasally; ns, not significant; pfu, plaque-forming units; preF, prefusion conformation–stabilized RSV F protein; RSV, respiratory syncytial virus; SD, standard deviation TNF-α, tumor necrosis factor α; vp, viral particles.


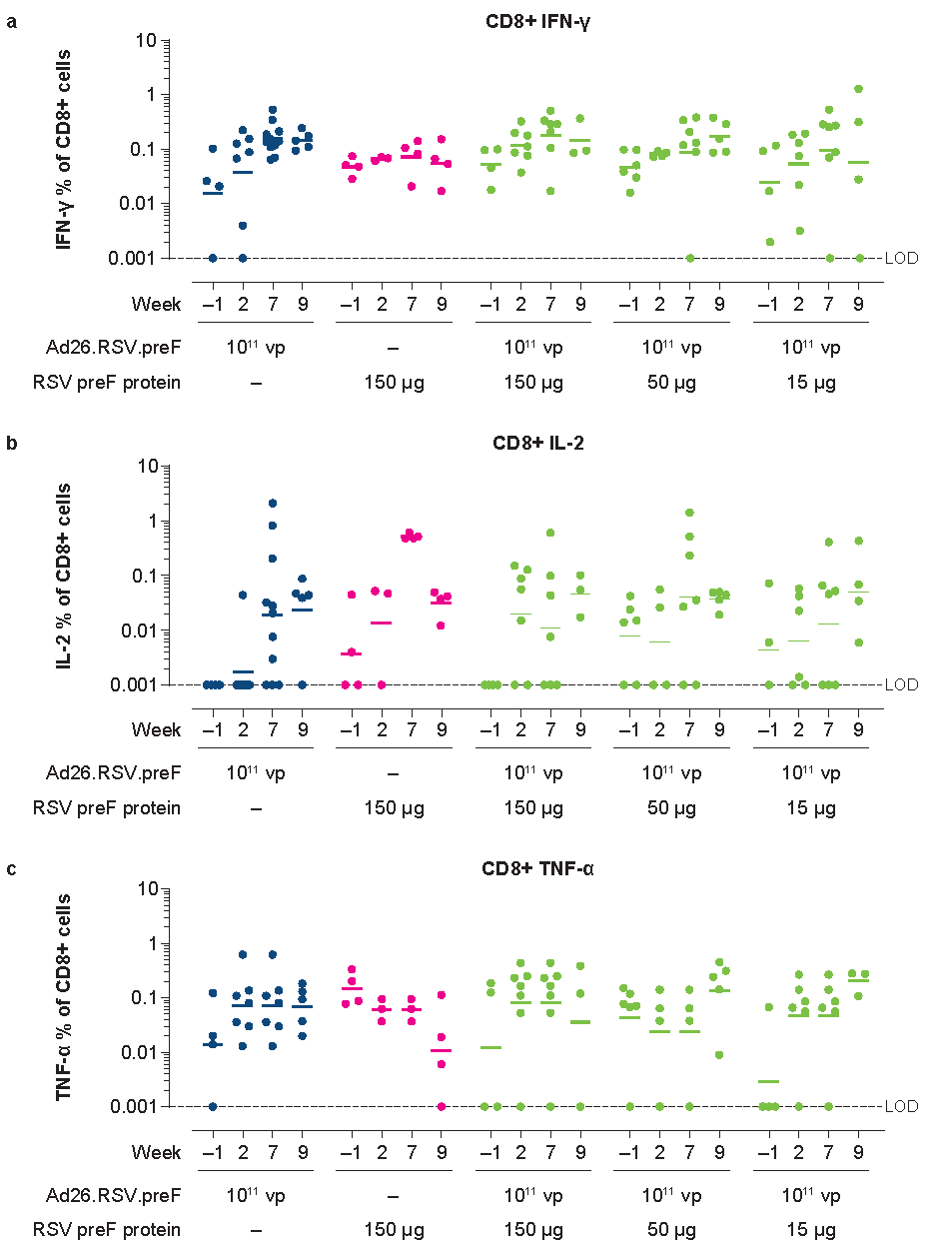


**Supplementary Figure 3. ICS measured from PBMCs isolated from African green monkeys.** Female African green monkeys were pre-exposed i.n. with RSV Memphis 37 (7.5×10^5^ pfu) at Week –19 and immunized intramuscularly with Ad26.RSV.preF (1×10^11^ vp; n = 11); RSV preF protein (150 µg; n = 4); or a combination of Ad26.RSV.preF (1×10^11^ vp) with RSV preF protein (150 µg; n = 7), RSV preF protein (50 µg; n = 7), or RSV preF protein (15 µg; n = 7) at Week 0. PBMCs were obtained before immunization and after immunization at Weeks 2, 7, and 9 for measurements of cellular immune responses by ICS. The percentage of CD8+ T cells expressing IFN-γ, TNF-α, and IL-2 in PBMCs of individual animals is shown. Horizontal bars indicate geometric mean responses. Samples with undetectable cytokine expression were set at LOD, depicted by a dotted line. Ad26, adenovirus type 26; ICS, intracellular cytokine staining; IFN-γ, interferon-γ; IL-2, interleukin-2; i.n., intranasally; LOD, limit of detection; PBMC, peripheral blood mononuclear cell; pfu, plaque-forming units; preF, prefusion conformation–stabilized RSV F protein; RSV, respiratory syncytial virus; TNF-α, tumor necrosis factor α; vp, viral particles.


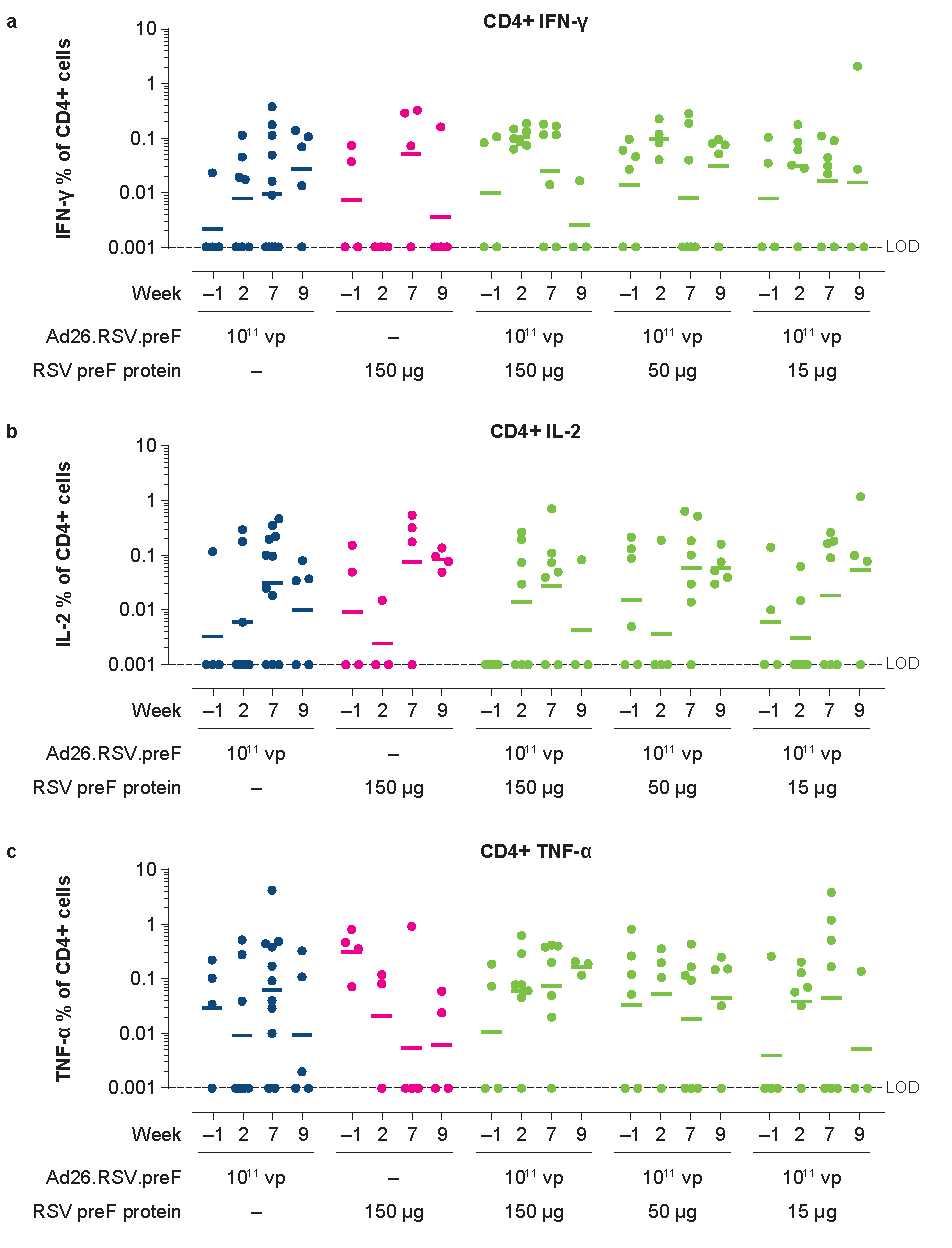


**Supplementary Figure 4. ICS measured from PBMCs isolated from African green monkeys.** Female African green monkeys were pre-exposed i.n. with RSV Memphis 37 (7.5×10^5^ pfu) at Week –19 and immunized intramuscularly with Ad26.RSV.preF (1×10^11^ vp; n = 11); RSV preF protein (150 µg; n = 4); or a combination of Ad26.RSV.preF (1×10^11^ vp) with RSV preF protein (150 µg; n = 7), RSV preF protein (50 µg; n = 7), or RSV preF protein (15 µg; n = 7) at Week 0. PBMCs were obtained before immunization and after immunization at Weeks 2, 7, and 9 for measurements of cellular immune responses by ICS. The percentage of CD4+ T cells expressing IFN-γ, TNF-α, and IL-2 in PBMCs of individual animals is shown. Horizontal bars indicate geometric mean responses. Samples with undetectable cytokine expression were set at LOD, depicted by a dotted line. Ad26, adenovirus type 26; ICS, intracellular cytokine staining; IFN-γ, interferon-γ; IL-2, interleukin-2; i.n., intranasally; LOD, limit of detection; PBMC, peripheral blood mononuclear cell; pfu, plaque-forming units; preF, prefusion conformation–stabilized RSV F protein; RSV, respiratory syncytial virus; TNF-α, tumor necrosis factor α; vp, viral particles.
