# Supplementary material for: Combination Ad26.RSV.preF/preF protein vaccine induces superior protective immunity compared with individual vaccine components in preclinical models
Source: NPJ Vaccines. 2023 Mar 23;8:45. doi: 10.1038/s41541-023-00637-7 (PMC10033289; doi:10.1038/s41541-023-00637-7)
Supplement: Supplementary file 2 — REPORTING SUMMARY [file 41541_2023_637_MOESM2_ESM.pdf]

nature portfolio

Corresponding author(s): Eirikur Saeland

Last updated by author(s): Feb 17, 2023

# Reporting Summary

Nature Portfolio wishes to improve the reproducibility of the work that we publish. This form provides structure for consistency and transparency in reporting. For further information on Nature Portfolio policies, see our Editorial Policies and the Editorial Policy Checklist.

## Statistics

For all statistical analyses, confirm that the following items are present in the figure legend, table legend, main text, or Methods section.

| n/a | Confirmed | |
|---|---|---|
| ☐ | ☒ | The exact sample size (*n*) for each experimental group/condition, given as a discrete number and unit of measurement |
| ☐ | ☒ | A statement on whether measurements were taken from distinct samples or whether the same sample was measured repeatedly |
| ☐ | ☒ | The statistical test(s) used AND whether they are one- or two-sided<br>*Only common tests should be described solely by name; describe more complex techniques in the Methods section.* |
| ☐ | ☒ | A description of all covariates tested |
| ☐ | ☒ | A description of any assumptions or corrections, such as tests of normality and adjustment for multiple comparisons |
| ☐ | ☒ | A full description of the statistical parameters including central tendency (e.g. means) or other basic estimates (e.g. regression coefficient) AND variation (e.g. standard deviation) or associated estimates of uncertainty (e.g. confidence intervals) |
| ☒ | ☐ | For null hypothesis testing, the test statistic (e.g. *F*, *t*, *r*) with confidence intervals, effect sizes, degrees of freedom and *P* value noted<br>*Give P values as exact values whenever suitable.* |
| ☒ | ☐ | For Bayesian analysis, information on the choice of priors and Markov chain Monte Carlo settings |
| ☒ | ☐ | For hierarchical and complex designs, identification of the appropriate level for tests and full reporting of outcomes |
| ☒ | ☐ | Estimates of effect sizes (e.g. Cohen's *d*, Pearson's *r*), indicating how they were calculated |

*Our web collection on statistics for biologists contains articles on many of the points above.*

## Software and code

Policy information about availability of computer code

| | |
|---|---|
| Data collection | All laboratory data were collected in Microsoft Excel. |
| Data analysis | Statistical analyses were performed in SAS version 9.4. |

For manuscripts utilizing custom algorithms or software that are central to the research but not yet described in published literature, software must be made available to editors and reviewers. We strongly encourage code deposition in a community repository (e.g. GitHub). See the Nature Portfolio guidelines for submitting code & software for further information.

## Data

Policy information about availability of data

All manuscripts must include a data availability statement. This statement should provide the following information, where applicable:

- Accession codes, unique identifiers, or web links for publicly available datasets
- A description of any restrictions on data availability
- For clinical datasets or third party data, please ensure that the statement adheres to our policy

All data to understand and assess the conclusions of this research are available in the main text and Supplementary Information. The raw data that support the findings of this study are available from the corresponding author upon reasonable request.

## Human research participants

Policy information about studies involving human research participants and Sex and Gender in Research.

| | |
|---|---|
| Reporting on sex and gender | No human research was performed |
| Population characteristics | No human research was performed |
| Recruitment | No human research was performed |
| Ethics oversight | No human research was performed |

Note that full information on the approval of the study protocol must also be provided in the manuscript.

# Field-specific reporting

Please select the one below that is the best fit for your research. If you are not sure, read the appropriate sections before making your selection.

☒ Life sciences ☐ Behavioural & social sciences ☐ Ecological, evolutionary & environmental sciences

For a reference copy of the document with all sections, see nature.com/documents/nr-reporting-summary-flat.pdf

# Life sciences study design

All studies must disclose on these points even when the disclosure is negative.

| | |
|---|---|
| Sample size | Sample size of the different mouse and cotton rats studies described in the manuscript are determined based on power calculations. Historical data for the investigated or similar vaccines were used to guide statistical powering. Power calculation was not used to determine the sample size in the non-human primate study. |
| Data exclusions | In the non-human primate study, data from nasal swabs that were contaminated with blood were excluded from analyses. |
| Replication | Samples from the different studies were generally analysed in a single experimental run, and reported as mean of 2-3 technical replicates. Individual runs were not repeated unless a documented technical issue in assay performance warranted re-analysis. |
| Randomization | Mice and cotton rats were randomized into the study groups at time of immunization. RSV pre-exposed non-human primates were were divided into 5 experimental groups, with comparable RSV F ELISA titers (measured 5 weeks before immunization), age distributions, and division between social groups. |
| Blinding | Staff involved in animal handling, treatment, sampling and observation were not blinded. Staff involved in data generation and analysis were not blinded. Blinding was not relevant for current studies as only objective quantitative measurements were performed. |

# Reporting for specific materials, systems and methods

We require information from authors about some types of materials, experimental systems and methods used in many studies. Here, indicate whether each material, system or method listed is relevant to your study. If you are not sure if a list item applies to your research, read the appropriate section before selecting a response.

### Materials & experimental systems

| n/a | Involved in the study | |
|---|---|---|
| ☐ | ☒ | Antibodies |
| ☐ | ☒ | Eukaryotic cell lines |
| ☒ | ☐ | Palaeontology and archaeology |
| ☐ | ☒ | Animals and other organisms |
| ☒ | ☐ | Clinical data |
| ☒ | ☐ | Dual use research of concern |

### Methods

| n/a | Involved in the study | |
|---|---|---|
| ☒ | ☐ | ChIP-seq |
| ☐ | ☒ | Flow cytometry |
| ☒ | ☐ | MRI-based neuroimaging |

## Antibodies

| | |
|---|---|
| Antibodies used | Antibody - Clone - Conjugation - Vendor - Catalog number<br>Goat anti-Mouse IgG-HRP (H+L) - polyclonal - HRP - Bio-Rad - 170-6516 |

Goat anti-Mouse IgG1 - polyclonal - HRP - SouthernBiotech - 1070-05
Goat anti-Mouse IgG2a - polyclonal - HRP - SouthernBiotech - 1080-05
Hamster anti mouse CD28 - 37.51 - NA - BD Biosciences - 553294
Rat anti-mouse CD49d - R1-2 - NA - BD Biosciences - 553153
Rat Anti mouse CD16/CD32 - 2.4G2 - NA - BD Biosciences - 553142
Hamster anti mouse CD3e - 145-2C11 - FITC - BD Biosciences - 553062
Rat anti mouse CD4 - RM4-5 - PerCpCy5.5 - BD Biosciences - 550954
Rat anti mouse CD8a - 53-6.7 - APC-H7 - BD Biosciences - 560182
Rat anti mouse IFN-g - XMG1.2 - PE - BD Biosciences - 554412
Rat anti mouse TNFa - MP6-XT22 - PE-Cy7 - BD Biosciences - 557644
Rat anti mouse IL2 - JES6-5H4 - APC - BD Biosciences - 554429
F(ab')2-Goat anti-Mouse IgG (H+L) – polyclonal - AF488 – Invitrogen - A-11017
Mouse Anti-Human CD4 - Clone L200 – FITC - BD Biosciences - 550628
Mouse Anti-Human IL-2 - Clone M7/48A - PE - Miltenyi Biotec - 130-091-646
Mouse Anti-Human CD14 - Clone TUK4 – PerCP - Miltenyi Biotec - 130-094-969
Mouse Anti-Human CD8 - Clone SK1 - PE-Cy7 – BioLegend - 344712
Mouse Anti-Human IFN-γ - Clone B27 – APC – BioLegend - 506510
Mouse Anti-Human CD3 - Clone SP34 - APC-Cy7 - BD Biosciences - 557757
Mouse Anti-Human TNF-α - Clone Mab11 - BV421 – BioLegend - 502932

Validation

All used antibodies are commercially available. Validation information is available on the manufacturers' websites.

## Eukaryotic cell lines

Policy information about cell lines and Sex and Gender in Research

Cell line source(s)

Chinese hamster ovary (CHO, hamster female) cells were originated from Lonza, a master cell bank was prepared, of which working banks were prepared. A549 cells (human male lung carcinoma) originated from the ATCC (ATCC® CCL-185™), a master cell bank was prepared, of which working banks were prepared. Hep-2 cells (human female cervical carcinoma, HeLa) originated the ATCC (ATCC® CCL-23™). Jurkat cells (human male lymphoma) stably transfected with mouse FcγRIV and NFAT-REluc2 expression plasmids originated from Promega, Madison, WI, USA. Peripheral blood mononuclear cells (PBMC) were derived from female african green monkeys.

Authentication

The cell lines were obtained as authenticated. No additional authentication was performed.

Mycoplasma contamination

Working cell banks were tested and found negative for Mycoplasma.

Commonly misidentified lines
(See ICLAC register)

Hep-2 cells (mentioned above).

## Animals and other research organisms

Policy information about studies involving animals; ARRIVE guidelines recommended for reporting animal research, and Sex and Gender in Research

Laboratory animals

Mouse studies: BALB/c mice, female, 6- to 8-weeks-old at the start of the study; cotton rat studies: female, 6- to 8-weeks old at the start of the study; Non-human primates: females 9-26 years of age.

Wild animals

The study did not involve wild animals.

Reporting on sex

Studies were only performed in female animals. For a careful comparison of induced immune responses between (combinations of) different vaccine candidates, as was the main objective of studies described in the current manuscript, it is important to keep the groups of experimental animals as homogeneous as possible. Therefore, experimental groups (in rodent experiments) were mostly comprised of inbred animals, from the same strain and age, as well as from the same sex. By reducing the variation, group sizes can be smaller, requiring fewer animals to address a specific research question. In the human population, more heterogenous immune responses can be expected. In addition to sex differences, many other factors (eg, genetic background, RSV pre-exposure history, underlying health conditions, and age) contribute to this heterogeneity. Therefore, in later stages of vaccine development, human testing in a heterogenous population comprised of both sexes is part of the vaccine development program.

Field-collected samples

Study did not involve samples collected from the field.

Ethics oversight

Mouse studies were conducted at Janssen Vaccines and Prevention B.V. according to the Dutch Animal Experimentation Act and the Guidelines on the Protection of Animals for scientific purposes by the Council of the European Committee after approval by the Centrale Commissie Dierproeven and the Dier Experimenten Commissie of Janssen Vaccines and Prevention B.V.
Cotton rat studies were conducted at Sigmovir Biosystems, Inc. by permission of the Institutional Animal Care and Use Committee (IACUC) of Sigmovir Biosystems, Inc.
The NHP study was conducted at the Wake Forest School of Medicine test facility and approved by the IACUC of Wake Forest University (WFU).

Note that full information on the approval of the study protocol must also be provided in the manuscript.

# Flow Cytometry

## Plots

Confirm that:

☒ The axis labels state the marker and fluorochrome used (e.g. CD4-FITC).

☒ The axis scales are clearly visible. Include numbers along axes only for bottom left plot of group (a 'group' is an analysis of identical markers).

☒ All plots are contour plots with outliers or pseudocolor plots.

☒ A numerical value for number of cells or percentage (with statistics) is provided.

## Methodology

| | |
|---|---|
| Sample preparation | For intracellular cytokine staining in mouse studies: After sacrifice, spleens (without attached fat tissue) were collected from the mice. The spleen was dissociated using the gentleMACS Dissociator (Miltenyi Biotec), or by manual grinding through a 70 µm cell strainer. Cells were centrifuged and treated with ACK lysis buffer (Lonza) for 2 minutes at room temperature to lyse red blood cells. After washing and centrifuging, cells were passed through a 30 µm pre-separation filter, resuspended and counted using a Vi-cell (Beckman Coulter).<br>For intracellular staining in non-human primate studies: PBMCs were isolated from blood by gradient centrifugation using Lympholite. Isolated PBMC were washed and cryopreserved. After thawing, cell counting an viability were determined using a Vi-cell. |
| Instrument | For both mouse and non-human primate studies a FACSCanto II (BD Biosciences) was used. |
| Software | BD FACSDiva software was used to acquire data. Further data analysis was performed using FlowJo software (mouse study) or DIVA software (non-human primate study). |
| Cell population abundance | No cell sorting was performed. |
| Gating strategy | For intracellular cytokine staining for the mouse study: Lymphocytes were identified on forward and side scatter (FSC and SSC) characteristics after exclusion of doublets based on their FSC-area/FSC-height characteristics, and exclusion of dead cells based on positivity for the Live/Dead cell discrimination fluorochrome (amine reactive dye). Cells were gated based on expression of CD4 or CD8 (note mouse cells were not gated based on CD3 high expression, as downregulation of CD3 is observed in activated T cells). Gates for the expression of IFNγ, TNFα, or IL-2 were based on fluorescence-minus-one controls (FMO-controls).<br>For intracellular staining for the non-human primate study: Lymphocytes were identified using a similar strategy as described for the mouse study, with the exception that CD14 cells were excluded and CD4 and CD8 T cells were gated within the CD3+ population. Gates for the expression of IFNγ, TNFα, or IL-2 were based on FMO-controls. |

☐ Tick this box to confirm that a figure exemplifying the gating strategy is provided in the Supplementary Information.
